# Developing quantitative analysis program of blood flow velocity according to vessel diameter for neovascular age-related macular degeneration using OCTA-VISTA

**DOI:** 10.1038/s41598-024-67271-8

**Published:** 2024-07-16

**Authors:** Fumi Tanaka, Toshihiro Mino, Yoshikiyo Moriguchi, Hidenori Nagahama, Masato Tamura, Yuji Oshima, Masahiro Akiba, Hiroshi Enaida

**Affiliations:** 1 Department of Ophthalmology, Faculty of Medicine, Saga University, 5-1-1 Nabeshima, Saga, 849-8501 Japan; 2 Research & Development Division, Topcon Corporation, 75-1 Hasunuma-Cho, Itabashi-Ku, Tokyo 174-8580 Japan; 3 Section of Ophthalmology, Department of Medicine, Fukuoka Dental College, 2-15-1 Tamura, Sawara-Ku, Fukuoka, 814-0193 Japan

**Keywords:** Neovascular age-related macular degeneration, Flow parameter, Vessel diameter, Optical coherence tomography angiography, Variable interscan time analysis, Diagnostic markers, Imaging and sensing

## Abstract

This study aimed to develop a quantitative analysis program of blood flow velocity by vessel diameter in neovascular age-related macular degeneration (nAMD) subjects using high-speed swept-source optical coherence tomography angiography. This retrospective, observational, cross-sectional study included 10 eyes of healthy volunteers and 4 eyes of patients with representative nAMD. Novel scan patterns and variable interscan time analysis were utilized to measure the flow parameter, a surrogate marker of blood flow velocity, by vessel diameter within different depths. Detected vessels at superficial and deep as well as outer retinal regions were categorized into three vessel diameters (major vessels (> 40 μm), medium vessels (20*–*40 μm), and capillaries (< 20 μm)). The flow parameter increased with enlarged vessel diameter in all participants at superficial and deep layer. All nAMD subjects, except for type 3 macular neovascularization (MNV), contained a structure dominated by medium vessels at outer retinal region. The mean flow parameter at outer retinal region was type 1 MNV (1.46 ms^−1^), type 1 + 2 MNV (0.98 ms^−1^), and polypoidal choroidal vasculopathy, including branching vascular networks (1.46 ms^−1^). This program provides the possibility to extract the blood flow information at different depths by vessel diameter types, which is considered to be useful tool for evaluating nAMD pathology and activity.

## Introduction

Evaluating microvascular blood flow is crucial in diagnosing various retinochoroidal disorders, including neovascular age-related macular degeneration (nAMD). Optical coherence tomography angiography (OCTA) is a procedure that visualizes vascular structures by contrasting motion-induced signal changes^1^. OCTA allows for rapid, noninvasive, and quantitative vascular structure evaluations. Spectral-domain (SD)-OCTA or swept-source (SS)-OCTA has provided even better macular neovascularization (MNV) visualization compared to indocyanine green angiography (ICGA)^2^. A study revealed that five structural features observed on OCT (intraretinal fluid, subretinal hyperreflective material, drusen, outer retinal tabulation, and hyperreflective foci) were associated with disease progression in nAMD^3^. Another study indicated that quantitative features obtained with OCTA, such as nAMD lesion size and morphological complexity (fractal dimension), are associated with disease activity^4–14^. Furthermore, morphological pattern changes in nAMD that occur during anti-vascular endothelial growth factor (VEGF) treatment in OCTA were found as disease activity biomarkers^15,16^. OCTA characterized the vascular structure remodeling associated with vascular tree branch reduction as the emergence of large-diameter vessels, the loss of small capillaries, and the marked anastomosis between vessels. Spaide proposed it as “arteriogenesis,” which is an abnormalization of vessels in the treated nAMD^17^. Therefore, identifying OCT/OCTA biomarkers of nAMD disease activity may guide treatment planning with anti-VEGF and is particularly important in managing patients with stable disease activity in treatment and extension plans.

Choi et al. and Moult et al. studied nAMD with a high A-scan rate SS-OCTA, quantitatively describing vessels in en-face OCTA with two different interscan times^18,19^. Later, Ploner et al*.* introduced the concept of variable interscan time analysis (VISTA) as a novel extension modality of OCTA^20^. More recently, Hwang et al*.* introduced a temporal autocorrelation decay model to VISTA, enabling the quantification of blood flow velocity using a surrogate marker^21^. In VISTA, the blood flow velocity marker is derived from the temporal changes in OCTA signals obtained at various time intervals, considering that OCTA signals exhibit more rapid changes with increased blood flow velocity. Incorporating information on blood flow velocity into the vascular network depicted by general OCTA facilitates comprehensive monitoring of disease progression^22^ However, previous studies that used VISTA revealed that the field of view was sometimes too narrow to depict the entire image of nAMD lesions. Additionally, motion artifacts sometimes prevent reliable analysis. Furthermore, previous studies have not performed a detailed evaluation of blood flow velocities classified by vessel diameter, including small vessel diameters, probably due to the lack of transverse resolution. Evaluating the activity of small vessels that comprise nAMD is considered important, particularly in anti-VEGF treatment, and analyzing metrics for each vessel diameter will demonstrate the significance of the blood flow velocimetry.

Obtaining a large amount of biological information about blood vessels is extremely important for identifying the pathological condition and the effectiveness of treating nAMD, which forms a complex vascular network. Additionally, obtaining such information non-invasively could be of great benefit to patients.

In this study, we employed a high-speed SS-OCT prototype with enhanced lateral resolution for the detailed delineation of capillaries which also enables the eye tracking free imaging with novel scanning method for wide-field VISTA. We develop a new nAMD blood flow evaluation program that enables to visualize and quantify the blood flow velocity in lesions of typical nAMD as a pilot study.

## Methods

This retrospective, cross-sectional, observational study obtained approval from the Institutional Ethics Committees of the Saga University Hospital and the Institutional Review Board of the Saga University Hospital (2021–05-07) and was performed under the ethical standards laid down by the Declaration of Helsinki. All participants signed written informed consent for the research and publication of this study and any accompanying images.

### Participants

All participants recruited in the Saga University Hospital, from November 2021 through August 2023, underwent comprehensive ophthalmologic examinations, including best-corrected visual acuity testing, slit-lamp examination, fundus examination, and color fundus photography, in addition to refraction testing and SS-OCT. Healthy controls were selected from patient volunteers with no ocular disease history, high myopia of -6.0 D or greater, or diabetes diagnosis. Additionally, we excluded eyes with poor OCTA image qualities due to cataracts, vitreous hemorrhage, or poor fixation. The four patients were diagnosed with nAMD by fluorescein angiography and ICGA.

Two retina specialists (FT and HN) investigated all ophthalmic examinations in this study. Additionally, a certified orthoptist (SY) performed SS-OCT measurements for all participants.

### Data acquisition and volume data reconstruction

This study used a high-resolution 400-kHz SS-OCT prototype^23^. The center wavelength of the swept source was 1050 nm with a tuning range of approximately 100 nm, which results in an 8-μm axial resolution. The incident beam diameter was expanded to 3 mm at the pupil plane, and astigmatism of the measured eye was corrected using a variable cross-cylinder lens, resulting in an approximately 5.5-μm transverse resolution.

The macular region of 7 mm in diameter was imaged using our scanning pattern called ammonite-scan^24^. Ammonite-scan consists of a fast circle B-scan and slow drift along a spiral trajectory. The circle perimeter was set to 4.5 mm and 512 A-lines data were acquired along the scan. This resulted in a 1.28-ms fundamental interscan time. The circle scan was repeated to get multiple-frame images necessary for OCTA and VISTA processing, and then the center of the circle was moved to the next position along the spiral trajectory toward the periphery. The circle scan repetition was set to five times, considering the balance between image quality and total acquisition time. The spiral drift perimeter was set to 54 mm in this study. Data acquisition was completed when the five repeated circle scans were performed at 2048 different locations along the spiral trajectory. The total acquisition time was approximately 13 s.

The volume acquired using an ammonite-scan was reconstructed in post-processing, using a previously reported method^25^. Briefly, motion during the measurement was estimated based on the correlation among the data collected at the same position but at different times. Motion-corrected volume data from OCT and decorrelation-based OCTA were reconstructed following the estimated motion. Here, the OCTA image was generated using the complex correlation mapping optical coherence angiography (cmOCA) algorithm^26^ with a spatial kernel size of 1 × 3 along the x × z direction.

### Vessel segmentation and diameter analysis

A layer segmentation was performed by applying the Topcon advanced boundary segmentation algorithm to the reconstructed OCT volume data^25^. Two retina specialists (FT and HN) checked the automated segmentation results and manually corrected them if needed. The superficial layer was the layer between the inner limiting membrane (ILM) and 15.6 μm below the inner nuclear layer (INL), while the deep layer was that between 15.6 μm and 70.2 μm below the INL. The enface projection was generated by maximum intensity projection within the slab. A vessel segmented image was analyzed to perform quantitative flow analysis with extraction of vessel area. Optimally oriented flux (OOF)^27^ response was first calculated for noise reduction at each pixel by changing the size of the OOF filter from 8 μm to 80 μm to get the vessel mask. Global and local thresholding was then applied to the OOF response image to generate a vessel mask^28^.

Vessel diameter was identified in the OOF calculation process as diameter maximizing the OOF response. The analyzed diameter was calibrated by considering the point spread function of the prototype. We generated a vessel diameter map for better visualization of the analyzed vessel diameter, where gray, green, and magenta represent capillaries (diameter < 20 μm), medium vessels (diameter ≥ 20 < 40 μm), and major vessels (diameter $\ge$ 40 μm), respectively. Based on the aforementioned vessel mask and vessel diameter map, the percentage of vessel area was calculated for each vessel diameter group. This program enables detailed retinal blood vessel and nAMD analysis by blood vessel diameter.

### VISTA

OCTA decorrelation signal change over time (t) was fitted under the model^21^, considering autocorrelation exponential decay and OCTA signal saturation as below.

$$Decorrelation\left( t \right) = D\left\{ {1 - \exp \left( { - \frac{t}{\tau }} \right)} \right\}$$

where D is the OCTA signal saturation level and $1/\tau$, the inverse of the time constant, which has a dimension of 1/time, was used as a surrogate marker of blood flow velocity (hereinafter referred to as “flow parameter”). A false-colored OCTA image (VISTA image) was then generated by superimposing the flow parameter onto the OCTA image.

### Quantification of flow properties within the region of interest (ROI)

The ROI for the retina was set as a circular area of 5 mm in diameter, centered on the fovea for all participants. Additionally, another ROI for the nAMD cases, except for type 3 MNV, was manually set to surround nAMD by checking the three-dimensional vascular connection by a skilled grader (TM) and approved by two retina specialists (FT, HN). The vessel mask and ROI were used to calculate vessel density and the mean flow parameter.

### Ethics declarations

This retrospective, cross-sectional, observational study obtained approval from the Institutional Ethics Committees of the Saga University Hospital and the Institutional Review Board of the Saga University Hospital (2021–05-07) and was performed under the ethical standards laid down by the Declaration of Helsinki. All participants signed written informed consent for the research and publication of this study and any accompanying images.

## Results

This study evaluated 10 healthy controls (6 males and 4 females) and 4 patients with nAMD (2 males and 2 females), with a mean age of 68.2 years (range: 58–80) and 74.8 years (range 72–77), respectively. The four nAMD cases were type 1, type 1 + 2, type 3 MNV, and polypoidal choroidal vasculopathy (PCV) based on MNV classification^29^. Three cases had an anti-VEGF multiple treatment history (Table 1).

**Table 1:** Participant demographics and characteristics.

	Number of eyes	Age, years	Gender	BCVA	Number of anti-VEGF treatment
Healthy controls	10	68.2 ± 6.8 (Range: 58–80)	Male:6 Female:4	20/16 (Range: 20/20 to 20/13)	0
Type 1 MNV	1	75	Male	20/33	28
Type 1 + 2 MNV	1	75	Male	20/22	27
Type 3 MNV	1	77	Female	20/22	0
PCV	1	72	Female	20/17	10

*BCVA* best-corrected visual acuity.

### Various OCTA and VISTA images of healthy controls

**Figure 1:**
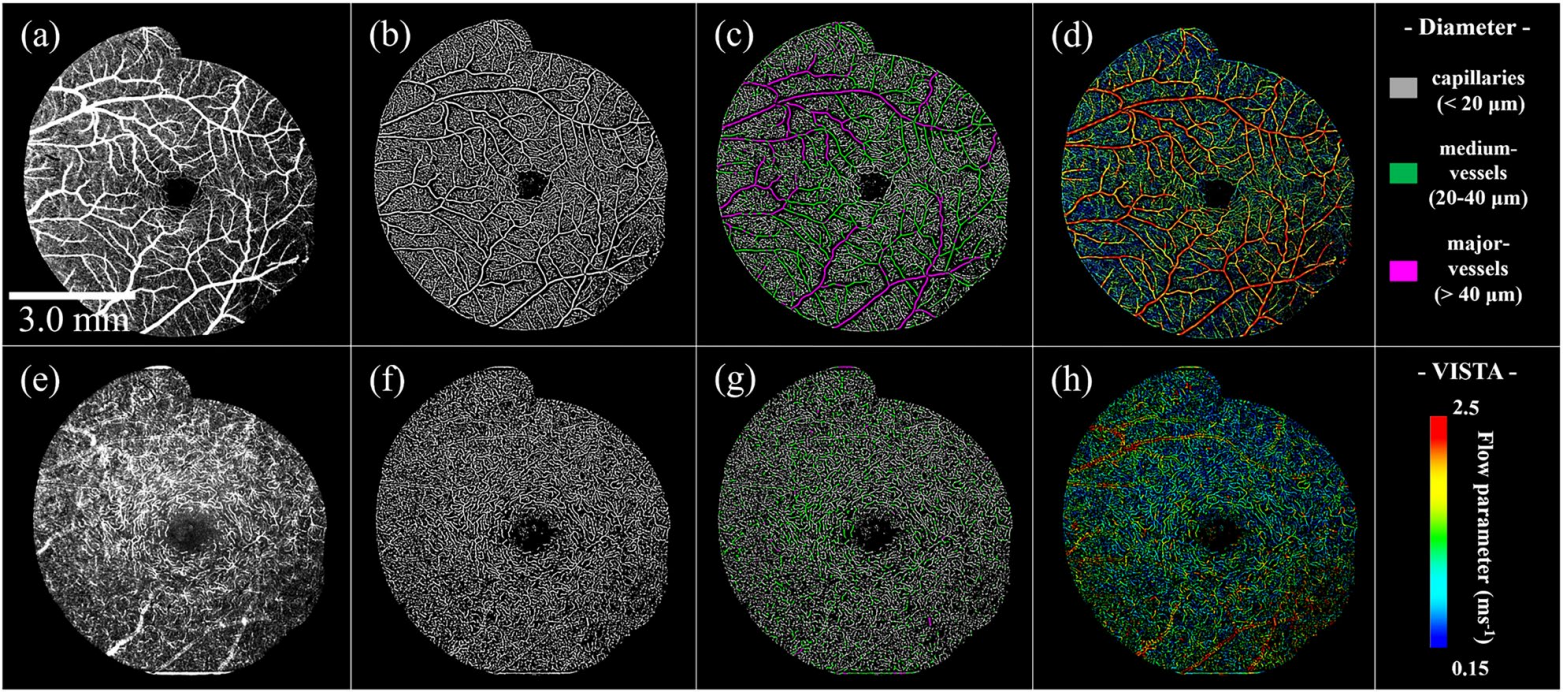
En-face images in a healthy control eye. (**a**) OCTA image of the superficial layer. (**b**) Vessel segmented image of the superficial layer. (**c**) Vessel diameter map of the superficial layer. Gray, green, and magenta locations represent capillaries, medium vessels, and major vessels. (**d**) VISTA image of the superficial layer. (**e**) OCTA image of deep layer. (**f**) Vessel segmented image of deep layer. (**g**) Vessel diameter map of deep layer. (**h**) VISTA image of deep layer.

Figure 1 shows an example of OCTA and VISTA images of the eyes of healthy controls acquired in this study. The OCTA image demonstrated successful visualization of the vascular structures of the superficial (Fig. 1a) and deep (Fig. 1e) layers in high definition over φ7 mm field-of-view in the macular center without motion artifacts. The vessel mask detected and visualized fine detail down to capillaries in both the superficial (Fig. 1b) and deep (Fig. 1f) retinal layer. The vessel diameter map of the superficial retinal layer (Fig. 1c) demonstrated major vessels coming directly from the optic disc in magenta (large), medium vessels in green (intermediate), and capillaries branching from the medium vessels in grey (small). In contrast, medium vessels (green) and capillaries (grey) were mainly observed in the deep layer (Fig. 1g), with only a few major vessels (magenta).

The vascular structure of the superficial retinal layer consisted of 67% ± 2% of capillaries, 24% ± 2% of medium vessels, and 9% ± 1% of major vessels. The vascular structure of the deep retinal layer consisted of 83% ± 3% of capillaries and 17% ± 3% of medium vessels (Table 2).

**Table 2:** Region of interest area, vessel density, and flow parameter for each vessel diameter in the retina of healthy controls and patients with nAMD.

		ROI area (mm^2^)	Vascular structure (%)				Flow parameter (ms^−1^)			
			Capillaries	Medium-vessels	Major-vessels	Total	Capillaries	Medium-vessels	Major-vessels	Mean
Healthy controls (n = 10)	Superficial retinal layer	19.63	67 ± 2	24 ± 1	9 ± 1	100	1.03 ± 0.15	1.80 ± 0.12	2.30 ± 0.02	1.33 ± 0.14
	Deep retinal layer		83 ± 3	17 ± 3	*	100	1.05 ± 0.19	1.20 ± 0.19	*	1.08 ± 0.19
nAMD (n = 4)	Superficial retinal layer	19.63	63 ± 5	23 ± 3	14 ± 3	100	0.91 ± 0.24	1.62 ± 0.25	2.25 ± 0.10	1.24 ± 0.25
	Deep retinal layer		83 ± 6	17 ± 5	*	100	0.97 ± 0.22	1.15 ± 0.15	*	1.00 ± 0.20
Type 1 MNV		1.29	26	45	29	100	0.94	1.57	1.75	1.46
Type 1 + 2 MNV		1.12	35	44	21	100	0.62	1.04	1.42	0.98
PCV		3.36	25	39	36	100	1.09	1.42	1.78	1.46

*Undetectable.

Major and medium vessels with fast blood flow were colored red to yellow, and capillaries with slow blood flow were colored green to blue in the VISTA image of the superficial layer (Fig. 1d). Medium vessels were colored green, and capillaries were colored green to blue in the deep layer (Fig. 1h). Mean flow parameters in the superficial layer were 1.33 ± 0.14 ms^−1^, 1.03 ± 0.15 ms^−1^, 1.80 ± 0.12 ms^−1^, and 2.30 ± 0.02 ms^−1^ for the entire ROI, capillaries, medium vessels, and major vessels, respectively. The mean flow parameters in the deep layer were 1.08 ± 0.19 ms^−1^, 1.05 ± 0.19 ms^−1^, and 1.20 ± 0.19 ms^−1^ for the entire ROI, capillaries, and medium vessels, respectively. The flow parameter was slower for smaller vessels and faster for larger vessels both in the superficial and deep retinal layers (Table 2).

### Verification of the association of retinal vessel diameter and velocity between normal and nAMD eyes

**Figure 2:**
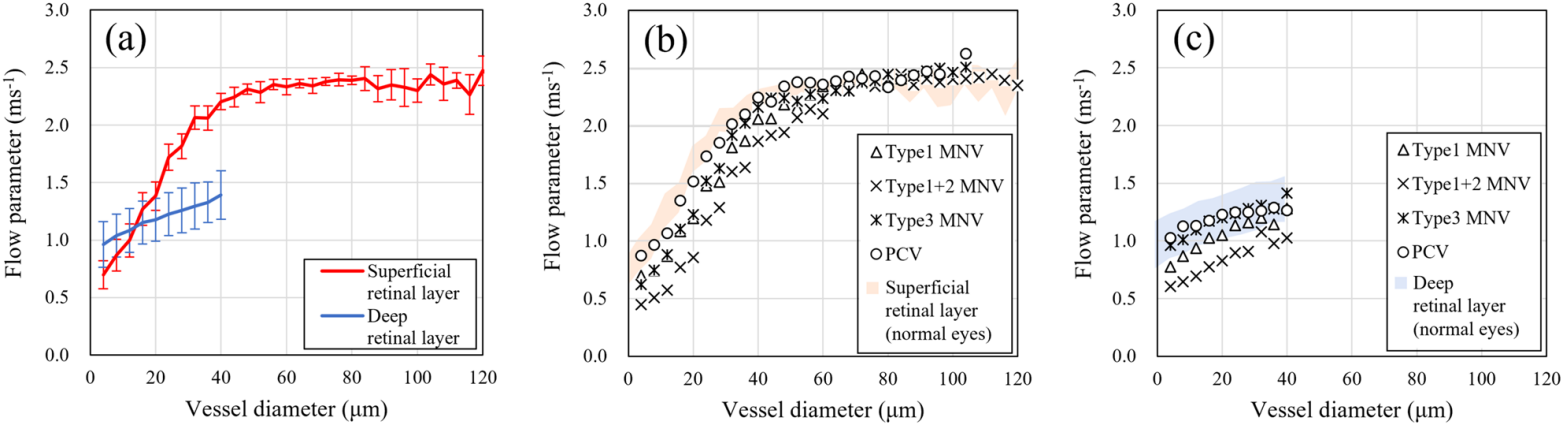
Association between vessel diameter and flow parameter. (**a**) Superficial retinal layer (red) and deep retinal layer (blue) of 10 healthy controls. The dots and bars indicate the average and standard deviation. (**b**) Superficial retinal layer of four patients with nAMD. The red area indicates the range of 10 healthy controls shown in (**a**). (**c**) The deep retinal layer of four patients with nAMD. The blue area indicates the range of 10 healthy controls shown in (**a**).

The association between vessel diameter and flow parameter in normal eyes indicated that the flow parameter linearly increased with vessel diameter, hitting a peak of approximately 2.5 ms^−1^ in the superficial retinal layer. Additionally, the flow parameter increases in the deep retinal layer as the vessel diameter expands, but the number of major vessels is extremely small and could not be measured (Fig. 2a). We analyzed flow parameter in nAMD cases in the same way as in normal eyes before the nAMD region. Plots of mean flow parameters in the superficial and deep layer of all patients with nAMD revealed relatively fast and slow flow parameters in the large vessels and small capillaries, respectively, in all cases. Additionally, a trend of reduced flow parameter in superficial and deep vessels was observed in nAMD cases compared to the normal eyes (Figs. 2b,c, Table 2).

### Various OCTA and VISTA images of representative nAMD cases

**Figure 3:**
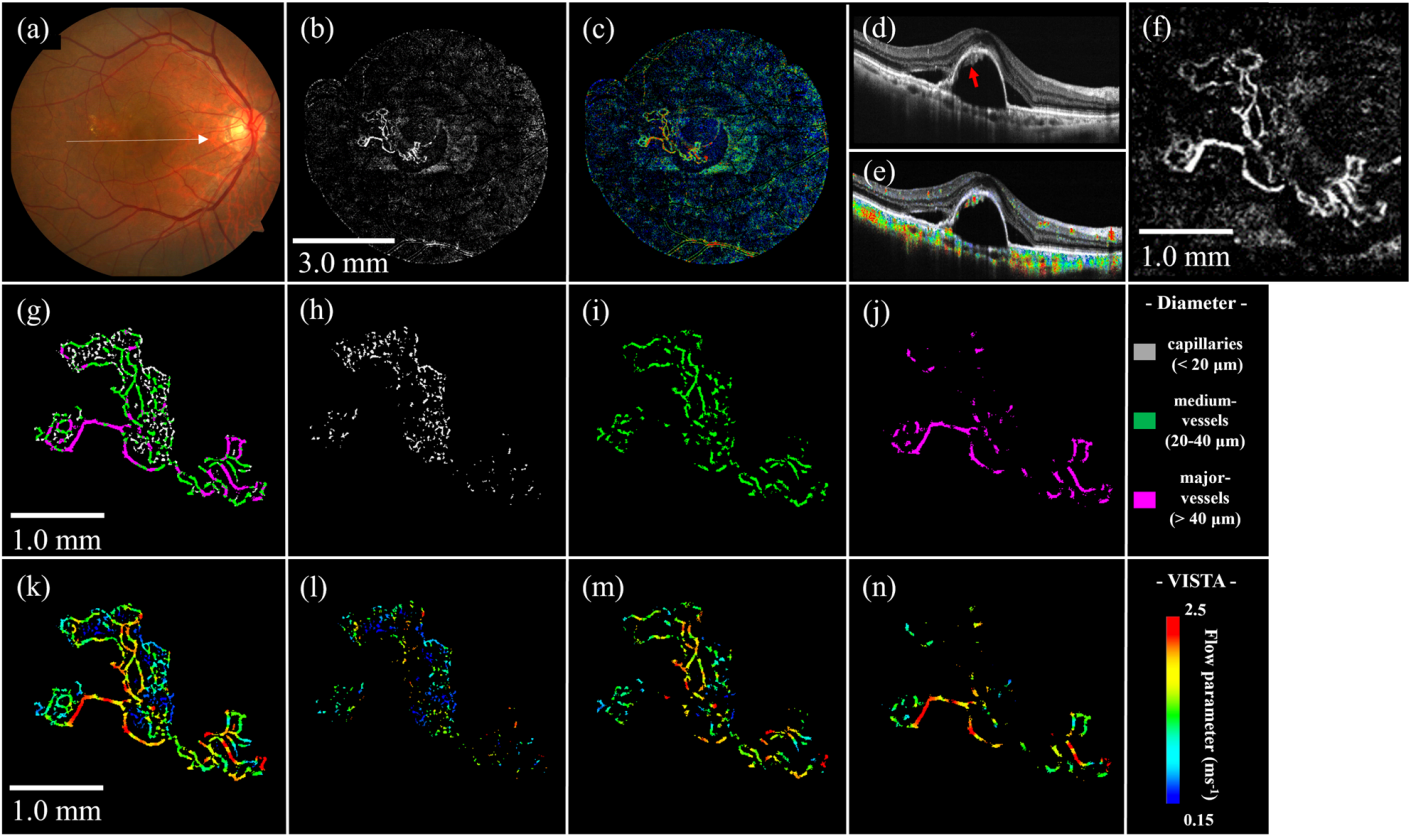
Type 1 MNV. (**a**) Fundus photograph. (**b**) Ammonite-scanning OCTA image of the outer retina. (**c**) VISTA image of the outer retina. (**d**) B-scan along the white arrow in (**a**). The red arrow indicates MNV, which is present along the RPE in the serous PED. (**e**) B-scan image with VISTA appended. (**f**) Enlarged view of the OCTA image (**b**) around the ROI. (**g**) Vessel diameter map in the ROI. (**h**–**j**) Vessel diameter maps with only capillaries, medium vessels, and major vessels extracted from the ROI. (**k**) VISTA image in the ROI. (**l**-**n**) Corresponding VISTA images.

The analysis of typical nAMD cases using the program mentioned above is presented. Color fundus photographs, as well as OCT/OCTA/VISTA images of type 1 MNV, are shown, respectively (Figs. 3a–f). MNV was clearly visualized by OCTA (Figs. 3b,f), and the B-scan image revealed MNV along the retinal pigment epithelium (RPE) in serous pigment epithelial detachment (PED), indicated by red arrows in Fig. 3d. The flow parameter of MNV inside the PED was clearly depicted on the VISTA (Fig. 3c), and B-scan with VISTA showed the presence of MNV blood flow along the PED (Fig. 3e). The structures of type 1 MNV were possible to resolve in three-vessel diameters (Figs. 3h–j). The vascular structure of this MNV consisted of 26% of capillaries, 45% of medium vessels, and 29% of major vessels (Table 2). The flow parameter in this type 1 MNV increases as the vessel diameter expands (Figs. 3g–n). Mean flow parameters were 1.46 ms^−1^, 0.94 ms^−1^, 1.57 ms^−1^, and 1.75 ms^−1^ for the entire ROI, capillaries, medium vessels, and major vessels, respectively (Table 2).

**Figure 4:**
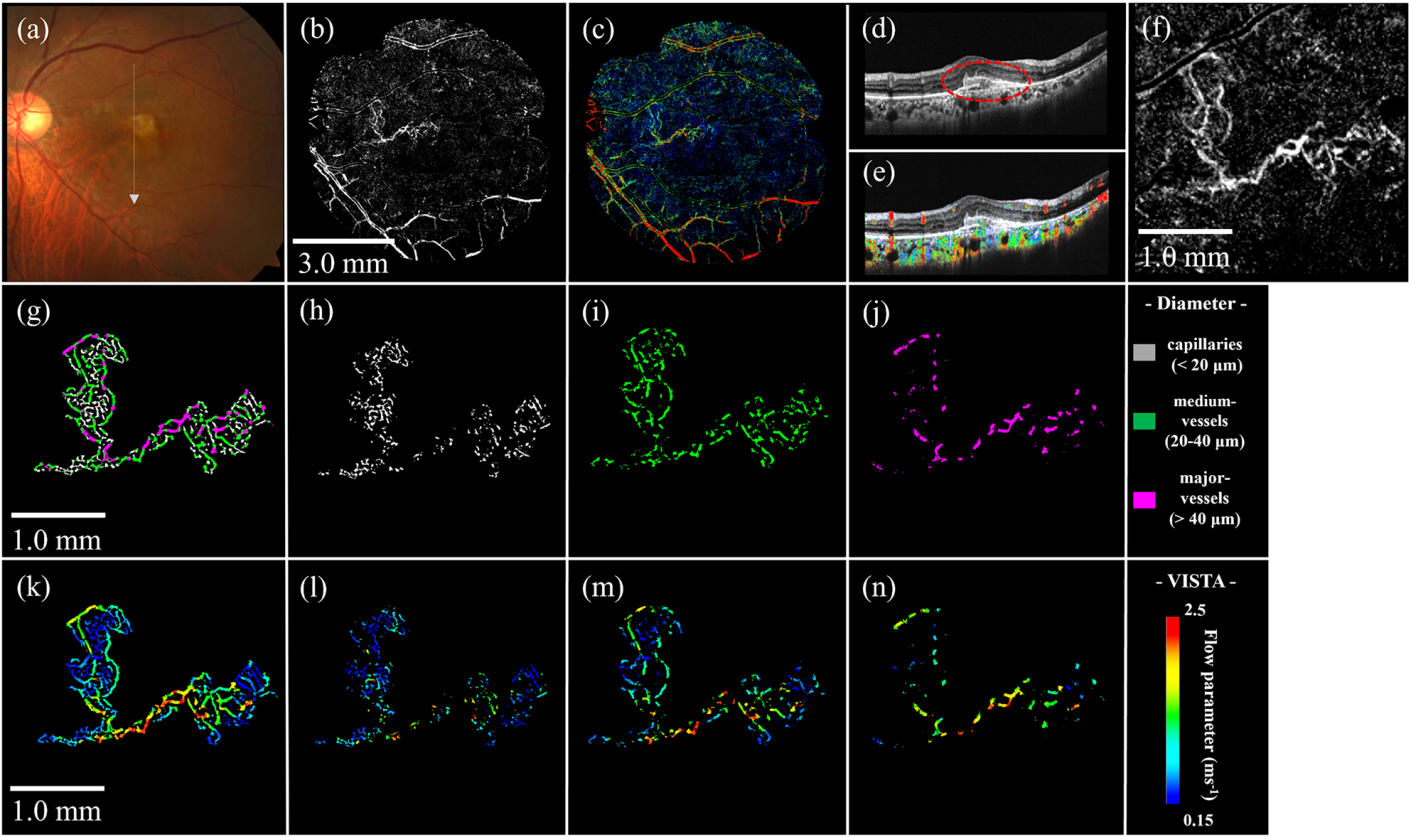
Type 1 + 2 MNV. (**a**) Fundus photograph. (**b**) Ammonite-scanning OCTA image of the outer retina. (**c**) VISTA image of the outer retina. (**d**) B-scan along the white arrow in (**a**). The dashed red circle indicates the MNV, which partially penetrates the RPE. (**e**) B-scan with VISTA appended. (**f**) Enlarged view of the OCTA image (**b**) around the ROI. (**g**) Vessel diameter map in the ROI. (**h**-**j**) Vessel diameter maps with only capillaries, medium vessels, and major vessels extracted from the ROI. (**k**) VISTA image in the ROI. (**l**-**n**) Corresponding VISTA images.

Consistent with the white lesion in the fundus photograph (Fig. 4a), MNV in type 1 + 2 MNV was clearly delineated by OCTA (Figs. 4b,f), and a complex of fibrin and the MNV was observed above and below the RPE as indicated by the red dashed line on the B-scan image (Fig. 4d). VISTA clearly depicted the flow parameter of MNV within the fibrin (Fig. 4c), while B-scan with VISTA more clearly depicted the progression of MNV over the RPE into the outer retinal layer (Fig. 4e). The vessel diameter map and VISTA by vessel diameter show that slow flow (blue) is more predominant in capillaries, while it is rarely seen in major vessels, similar to type 1 MNV. The flow parameter increases with expanding vessel diameter (Figs. 4g–n). The vascular structure of this MNV consisted of 35% of capillaries, 44% of medium vessels, and 21% of major vessels. Mean flow parameters were 0.98 ms^−1^, 0.62 ms^−1^, 1.04 ms^−1^, and 1.42 ms^−1^ for the entire ROI, capillaries, medium vessels, and major vessels, respectively (Table 2).

**Figure 5:**
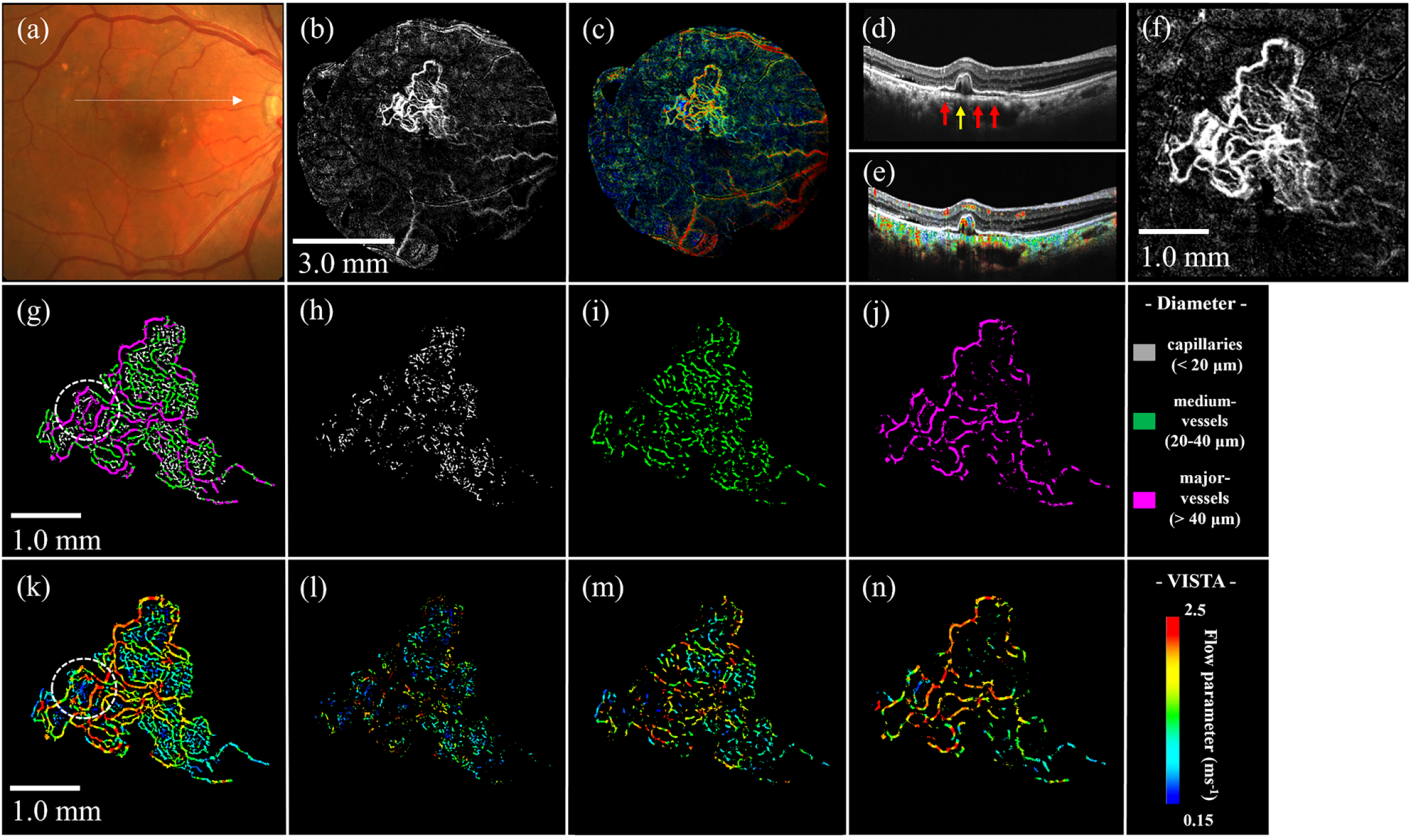
PCV. (**a**) Fundus photograph. (**b**) Ammonite-scanning OCTA image of the outer retina. (**c**) VISTA image of the outer retina. (**d**) B-scan along the position of the white arrow in (**a**). The yellow arrow indicates a polypoidal lesion and the red arrow represents BVN. (**e**) B-scan with VISTA appended. (**f**) Enlarged view of the OCTA image (**b**) around the ROI. (**g**) Vessel diameter map in the ROI. The white dashed circle indicates a polypoidal lesion. (**h**–**j**) Vessel diameter maps with only capillaries, medium vessels, and major vessels extracted from the ROI. (**k**) VISTA image in the ROI. (**l**-**n**) Corresponding VISTA images.

Fundus photograph in PCV showed reddish-orange polypoidal lesions (Fig. 5a), and OCTA clearly depicted MNV, but distinguishing between branching vascular network (BVN) and PCV was difficult (Fig. 5b). OCT B-scan image (Fig. 5d) exhibit BVN and a steeply elevated PED (polypoidal lesion) as indicated by red and yellow arrows, respectively. Blood flow in polypoidal lesions demonstrated a mixture of fast and slow sites in the VISTA image (Figs. 5c,e). Polypoidal lesions included large and small blood vessels, as well as a mixture of slow and fast flows as seen in the white dashed circle area in Figs. 5g,k. BVN, as well as types 1 and 1 + 2 MNV, show that flow parameter increases with expanding vessel diameter (Figs. 5g–n). The vascular structure of this PCV, including BVN, consisted of 25% of capillaries, 39% of medium-vessels, and 36% of major-vessels. Mean flow parameters were 1.46 ms^−1^, 1.09 ms^−1^, 1.42 ms^−1^, and 1.78 ms^−1^ for the entire ROI, capillaries, medium vessels, and major vessels, respectively (Table 2).

**Figure 6:**
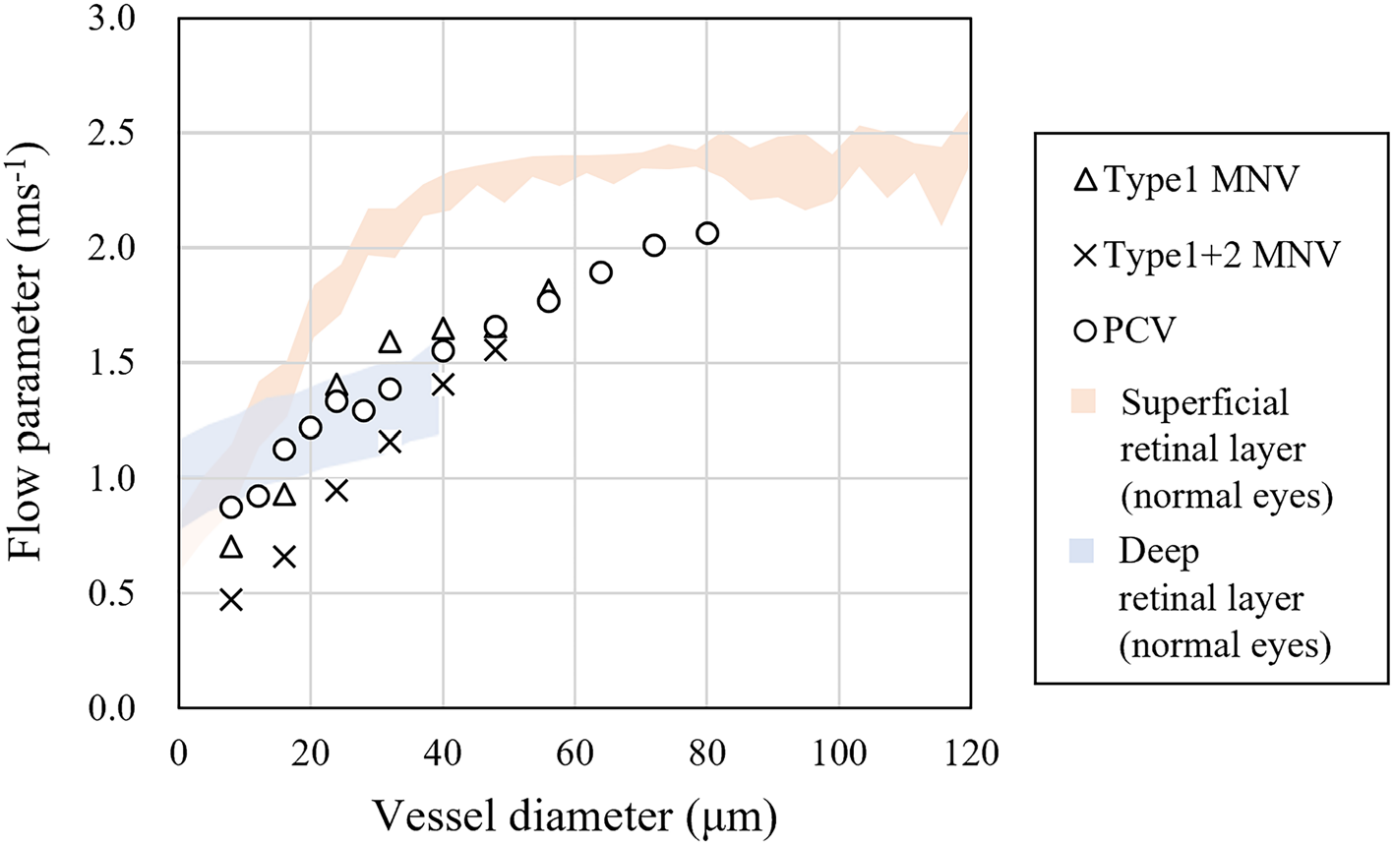
Association between vessel diameter and flow parameters of MNV. For comparison, the red and blue areas indicate the range of flow parameters from superficial and deep layers of the 10 healthy controls shown in Fig. 2a; flow parameters of MNV are closer to those of the deep layer than to those of superficial layer in healthy controls.

OCTA en-face images of nAMD are detailed without motion artifacts (Figs. 3–5). All of the nAMD cases, except for type 3 MNV, analyzed in this study demonstrated a structure dominated by medium vessels (Table 2). Moreover, the association between vessel diameter and velocity in nAMDs was more similar to that in the deep retina than in the superficial retina (Fig. 6). The lesions for type 3 MNV were extremely small and difficult to visualize with en-face images (Supplemental data). Therefore, VISTA and vessel diameter analysis was impossible.

## Discussion

Previous studies have confirmed the usefulness of OCTA in diagnosing and monitoring different nAMD types^2,7,11,14^. Since the advocacy of Spaide’s abnormalization theory of nAMD treated with recurrent intravitreal anti-VEGF injections^17^, different studies have focused on changes in nAMD blood vessels, especially retinal capillaries. In this study, we successfully evaluated the flow parameter of the normal retina and nAMD lesion for each of the three vessel diameters using a recently developed scanning protocol and high-speed, high-resolution OCT with the VISTA method. This is the first demonstration of a potential new biomarker, the mean flow parameter separately analyzed by the vessel diameter.

The flow parameters in the superficial and deep layers of normal eyes exhibited a linear association between vessel diameter and flow parameter (Fig. 2a). This is consistent with a previous report by Wang et al.^30^*.* The authors measured blood flow velocity in the vessel diameter from < 20 μm to > 100 μm using a retinal function imager on normal eyes. The results revealed that both arterioles and venules demonstrated a positive linear correlation with blood vessel diameter and velocity. Palochak et al*.* used an adaptive optics scanning laser ophthalmoscopy to measure arterial and venous blood flow velocities in superficial retinal vessels of 15–100 μm in diameter and revealed that the velocity increases with expanding diameter^31^. The results of studies using these other modalities are consistent with our findings and support the validity of the flow parameter outcomes obtained with VISTA.

Several requirements are required to ensure reliable nAMD assessment with VISTA, including (1) reduced motion artifacts^22^, (2) high optical/digital transverse resolution^21^, (3) short interscan time, (4) multiple repetitions, and (5) wide field-of-view. Additionally, tradeoffs were observed between them. In particular, improved optical transverse resolution will increase sensitivity to eye motions. The denser the digital transverse resolution, the wider the scanning area, or the greater the number of repetitions, the longer the total acquisition time will extend. Consequently, the image is also prone to contamination by eye motions. This is more problematic in cases of unstable fixation due to low central vision, such as eyes with nAMD. Conversely, extending the imaging area or improving digital transverse resolution is difficult while maintaining fundamental interscan time, which determines the detectable velocity range in the VISTA. The new scan pattern, called “ammonite scan,”^24^ used in this study efficiently solved these issues. Fast circle scans in the ammonite scan are repeated and moved to the periphery in a spiral manner with redundancy. The imaging area was decoupled from the sampling resolution and interscan time in this approach. Hence, the entire nAMD legion could be imaged while keeping a sufficient detectable velocity range so that velocity heterogeneity could be seen in the VISTA images. Furthermore, generating VISTA images with effectively reduced motion artifacts, which are seen from some of the images shown in the results, was possible by applying a motion compensation algorithm to the redundant data.

In this study, we visualized VISTA images of three representative cases of nAMD, each with three different vessel diameters. Quantifying the vascular structure and flow parameter has become possible at the lesion. This program enables the clarification of more detailed individualized hemodynamics of nAMD.

This study identified flow parameter in nAMDs as slower for smaller vessel diameters and faster for larger vessel diameters. The flow parameter in the MNV resembled that in the deep retina more than in the superficial retina of normal eyes. This is probably because the MNV exhibits a vascular structure with many tortuous and branching vessels, with few linear vessels like those on the superficial retina (Fig. 6, Table 2). Rebhun et al*.* used VISTA and revealed faster blood flow in larger vessels and slower blood flow in smaller vessels inside the MNV^22^, which is consistent with our findings. Notably, the patients with nAMD included in this study had already received multiple anti-VEGF treatments, and their vascular structure may have differed from that of treatment-naïve patients.

Establishing an ROI was difficult for vertically progressive type 3 MNV in this study, and it was excluded from the analysis. Previous studies revealed an association between high PED and OCTA undetectability^32,33^. Faatz et al*.* showed differences in the pathological vasculature of untreated MNV of types 1, 2, and 3 MNV, using SS-OCTA^34^. Type 3 MNV demonstrated a much smaller MNV area than the other types because the direction of angiogenic growth begins from the deep retinal vascular plexus to the RPE, axial blood flow. Capturing such longitudinal structures is difficult due to the nature of OCTA. The method constructed in this study may be more suitable for horizontally progressive nAMD, such as type 1 MNV, type 2 MNV, and BNV of PCV, in MNV quantification. Further cases need to be evaluated to investigate the hemodynamic characteristics of each type of nAMD.

Our results emphasize a future perspective that enables flow parameter evaluation by vessel diameter before and after treatment and during the course. Anti-VEGF treatment reduces finer vessels in the nAMD and concentrates blood flow in larger vessels^16,17,22^. The correlation between vessel diameter and blood flow velocity, reported in previous studies^30,31^, can be used as an indicator that vessels with high blood flow velocity after anti-VEGF treatment are mature vessels, while those with low blood flow velocity are immature vessels. Confirming the association of anti-VEGF treatment with flow parameters in capillaries, medium vessels, and large vessels will help in evaluating treatment efficacy. Incorporating vessel diameter as a new biomarker, in addition to nAMD area, in assessing flow parameter is expected to add more clinical value in evaluating disease activity, therapeutic agent efficacy, and treatment endpoints and resumption. Additionally, the combination with the B-scan imaging is expected to enhance the ability to determine disease activity and reduce patient burden by decreasing the number of treatment cycles. Further studies are warranted to investigate pre- and post-treatment changes and treatment efficacy to achieve these goals, and how these changes when there is marked response or resistance to treatment in untreated patients. Our future studies will investigate blood flow changes before and after anti-VEGF treatment in treatment-naïve patients.

## Limitations

This study aimed to develop a new nAMD blood flow evaluation program sample as a pilot study. As a limitation, the small sample size limited the statistical evaluation of the proportion of vessel density or flow parameter for each nAMD type.

Next, the exponential decay model proposed by Hwang et al*.*^21^ was used to quantify blood flow velocity. Although the use of this model is reasonable, it represents one of several possible models for quantifying blood flow velocity. Alternative models such as those proposed by Lee et al*.* and Tokayer et al*.*^35,36^, may offer more precise descriptions of blood flow velocity. Additionally, the flow parameter measured using the exponential decay model served as a surrogate marker for velocity rather than a direct measurement. This metric provides quantitative data with dimensions of 1/time, offering an advantage over the dimensionless “relative blood flow velocity” derived from the original VISTA^20^. However, this is distinct from the absolute velocity measured in adaptive optics studies^37^, representing a limitation of the VISTA.

Then, the blood flow in nAMD could be visualized and evaluated similarly to retinal blood flow using VISTA, but analysis of choroidal blood flow is difficult even with SS-OCT, which has a higher penetration depth. The OCT signal strength in the choroidal vessels, especially in the Haller layer, is generally weak due to light absorption and scattering in RPE and choriocapillaris, fringe wash-out, and thresholding used in signal processing^1,38^. Interestingly, an OCTA signal was seen in the choroidal stroma, the origin of this signal is obscure at this point and must be interpreted and evaluated with caution.

### Supplementary Information

Supplementary Information.

## Data Availability

The datasets analyzed in the current study are available from the corresponding author upon request.
