# Supplementary material for: Developing quantitative analysis program of blood flow velocity according to vessel diameter for neovascular age-related macular degeneration using OCTA-VISTA
Source: Sci Rep. 2024 Jul 16;14:16352. doi: 10.1038/s41598-024-67271-8 (PMC11252384; doi:10.1038/s41598-024-67271-8)
Supplement: Supplementary file 1 — Supplementary Information. [file 41598_2024_67271_MOESM1_ESM.pdf]

Developing quantitative analysis program of blood flow velocity according to vessel diameter for neovascular age-related macular degeneration using OCTA-VISTA

Fumi Tanaka, Toshihiro Mino, Yoshikiyo Moriguchi, Hidenori Nagahama, Masato Tamura, Yuji Oshima, Masahiro Akiba, and Hiroshi Enaida

Supplementary information

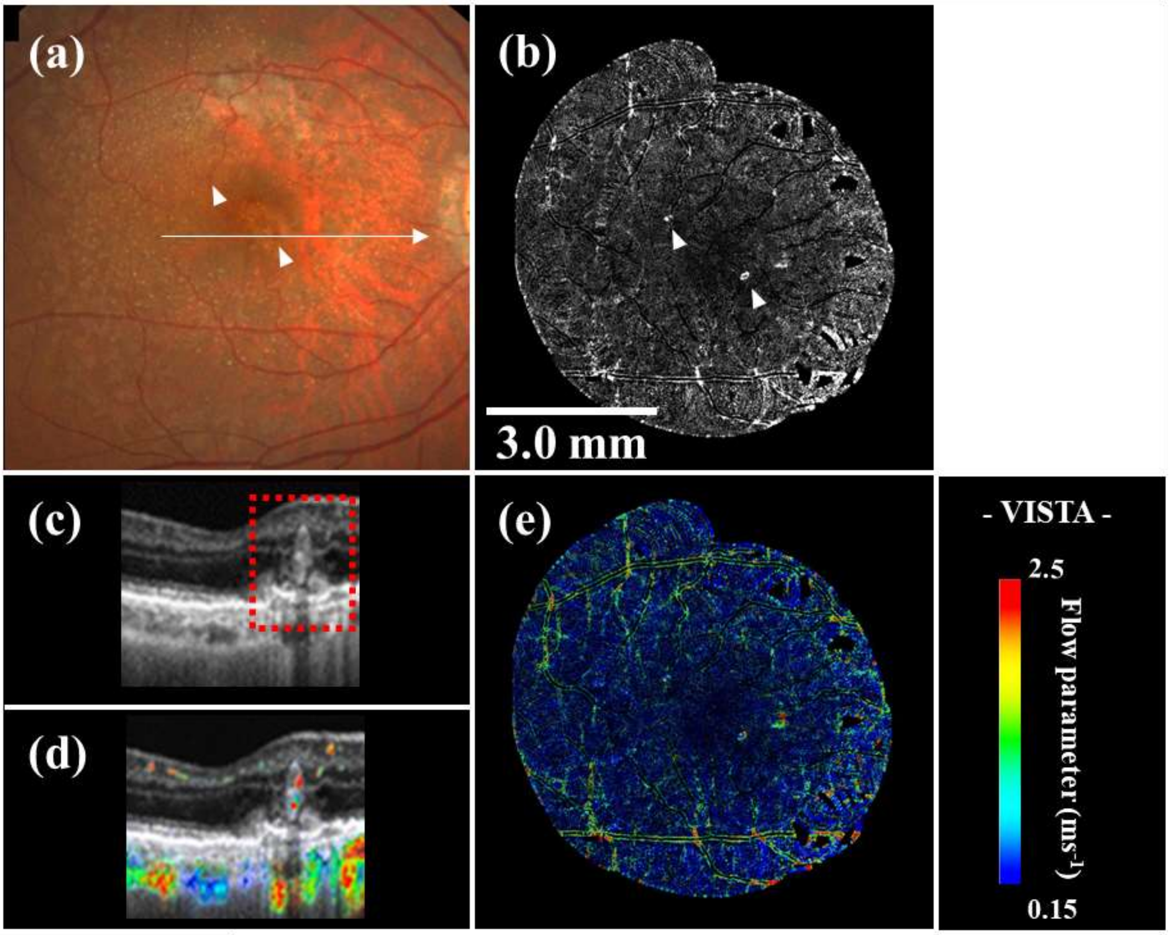

The fundus photograph shows diffuse drusen and dot hemorrhages in type 3 macular neovascularization (MNV) indicated by white arrowheads (a), and the dot hemorrhages were visualized as MNV in the optical coherence tomography angiography (OCTA) image (b). Intraretinal neovascularization (IRN) has progressed under the retina and the retinal pigment

epithelium (RPE) line has become irregular, as indicated by the red dashed square in the B-scan image (c). Visualization of the blood flow signal in IRN confirmed that RPE was not perforated in the B-scan with variable interscan time analysis (VISTA) (d). Flow parameters of MNV were depicted by VISTA (e) but were excluded from the subsequent quantitative analysis because the MNV area was extremely small in the enface image and it was difficult to set the region of interest (ROI).
